# Automatized FACEmemory® scoring is related to Alzheimer’s disease phenotype and biomarkers in early-onset mild cognitive impairment: the BIOFACE cohort

**DOI:** 10.1186/s13195-022-00988-8

**Published:** 2022-03-18

**Authors:** Montserrat Alegret, Oscar Sotolongo-Grau, Ester Esteban de Antonio, Alba Pérez-Cordón, Adelina Orellana, Ana Espinosa, Silvia Gil, Daniel Jiménez, Gemma Ortega, Angela Sanabria, Natalia Roberto, Isabel Hernández, Maitee Rosende-Roca, Juan Pablo Tartari, Emilio Alarcon-Martin, Itziar de Rojas, Laura Montrreal, Xavier Morató, Amanda Cano, Dorene M. Rentz, Lluís Tárraga, Agustín Ruiz, Sergi Valero, Marta Marquié, Mercè Boada

**Affiliations:** 1 Ace Alzheimer Center Barcelona—Universitat Internacional de Catalunya, Gran Via de Carles III, 85 bis, 08028 Barcelona, Spain; 2 Networking Research Center on Neurodegenerative Diseases (CIBERNED), Instituto de Salud Carlos III, Madrid, Spain; 3 Center for Alzheimer Research and Treatment, Department of Neurology, Brigham and Women’s Hospital, Boston, MA USA; 4 Department of Neurology, Massachusetts General Hospital, Boston, MA USA

**Keywords:** Endophenotype, Early detection, Alzheimer’s disease, New technologies, Episodic memory, Computerized assessment, Cognitive impairment, Biomarkers, Brain magnetic resonance, Cerebrospinal fluid

## Abstract

**Background:**

FACEmemory® is the first computerized, self-administered verbal episodic memory test with voice recognition. It can be conducted under minimal supervision and contains an automatic scoring system to avoid administrator errors. Moreover, it is suitable for discriminating between cognitively healthy and amnestic mild cognitive impairment (MCI) individuals, and it is associated with Alzheimer’s disease (AD) cerebrospinal fluid (CSF) biomarkers. This study aimed to determine whether FACEmemory scoring is related to performance on classical memory tests and to AD biomarkers of brain magnetic resonance imaging (MRI) and CSF in patients with early-onset MCI (EOMCI).

**Methods:**

Ninety-four patients with EOMCI from the BIOFACE study completed FACEmemory, classical memory tests (the Spanish version of the Word Free and Cued Selective Reminding Test -FCSRT-, the Word List from the Wechsler Memory Scale, third edition, and the Spanish version of the Rey–Osterrieth Complex Figure Test), and a brain MRI. Eighty-two individuals also underwent a lumbar puncture.

**Results:**

FACEmemory scoring was moderately correlated with FCSRT scoring. With regard to neuroimaging MRI results, worse execution on FACEmemory was associated with lower cortical volume in the right prefrontal and inferior parietal areas, along with the left temporal and associative occipital areas. Moreover, the total FACEmemory score correlated with CSF AD biomarkers (Aβ1-42/Aβ1-40 ratio, p181-tau, and Aβ1-42/p181-tau ratio). When performance on FACEmemory was compared among the ATN classification groups, significant differences between the AD group and normal and SNAP groups were found.

**Conclusions:**

FACEmemory is a promising tool for detecting memory deficits sensitive to early-onset AD, but it also allows the detection of memory-impaired cases due to other etiologies. Our findings suggest that FACEmemory scoring can detect the AD endophenotype and that it is also associated with AD-related changes in MRI and CSF in patients with EOMCI. The computerized FACEmemory tool might be an opportunity to facilitate early detection of MCI in younger people than 65, who have a growing interest in new technologies.

## Background

Alzheimer’s disease (AD) is the most prevalent neurodegenerative disorder causing dementia. It is characterized by a continuous decline in both cognitive and functional abilities along a spectrum from subjective cognitive decline to mild cognitive impairment (MCI) and dementia [1]. In only about 5% of cases do the first symptoms begin at less than 65 years of age, a condition known as early-onset Alzheimer’s disease (EOAD) [2].

The diagnosis of early-onset MCI (EOMCI) is more complicated, probably due to a lower suspicion of neurodegenerative conditions in younger people and a broad differential diagnosis [2]. This prodromal stage of dementia in these younger individuals is, in parallel, where cognitive deficits are already evident on formal neuropsychological testing although autonomy in their activities of daily living is preserved [3]. EOMCI is a distinct category of MCI not always related to prodromal AD, as many other underlying conditions, such as Parkinson’s disease or depression, can be associated with this entity [4]. Thus, there is a need for newer diagnostic tools to detect the first signs of AD in EOMCI.

It is well known that one of the endophenotypes proposed for AD is episodic memory disturbances [5]. Some studies report that EOAD patients have more difficulty learning new information that is not recalled with clues, reinforcing the hypothesis that a more severe storage or hippocampal memory impairment pattern exists in the younger group [6, 7]. However, other studies suggest that EOAD may begin with an atypical non-amnestic pattern [8, 9].

The International Working Group [10] recommends the use of the Free and Cued Selective Reminding Test (FCSRT) [11] to assess episodic memory. FCSRT is effective in detecting amnestic MCI and dementia [12], with total recall impairment being the core clinical phenotype of prodromal AD [13]. Moreover, FCSRT has been found to be useful in predicting conversion from MCI to AD dementia [14], and it is associated with AD neuroimaging [15] and cerebrospinal fluid (CSF) biomarkers [16]. However, this test requires a reliable and highly trained professional to administer it.

As a result of the growing interest in developing newer technological tools for detecting the first signs of AD prior to the MCI stage, we recently developed and validated the FACEmemory® test [17]. This is a computerized version of the abbreviated form [18] of the original Face-Name Associative Memory Exam (FNAME) [19], a complex episodic memory test developed by Dr. Rentz’s team [18] to detect preclinical [20] and prodromal AD. FACEmemory is unique in that it is the first self-administered verbal memory test with voice recognition and conducted under minimal supervision. It has an automatic scoring system to avoid administrator error. Moreover, it is suitable for discriminating between cognitively healthy and amnestic MCI (aMCI) individuals, who in most cases convert to AD [21, 22], and it is correlated with AD CSF biomarkers [17].

The work presented herein is part of the BIOFACE project, a longitudinal study based on a cohort of 97 patients with EOMCI and focused on increasing the knowledge of AD’s prodromal stages, including the clinical and biomarker characterization of EOMCI [23]. The main aim of the present study is to determine whether FACEmemory scoring is related to performance on classical memory tests and to AD biomarkers of brain magnetic resonance imaging (MRI) and CSF in patients with EOMCI. We hypothesized that worse performance on FACEmemory would be associated with worse performance on the FCSRT and would be related to MRI and CSF AD biomarkers in EOMCI.

## Methods

### Participants

A total of 97 participants with EOMCI from the BIOFACE study [23] were recruited at the Memory Unit of Ace Alzheimer Center Barcelona. Three were excluded from analysis because they failed to complete the FACEmemory test (one) or whole MRI data was not available for quantitative analysis (two).

### Neurobehavioral assessment

As detailed previously [23], a comprehensive neuropsychological, neurological, and functional assessment at the Memory Unit of Ace Alzheimer Center Barcelona, as well as routine analyses of blood and structural brain neuroimaging, were administered to all BIOFACE participants within a time window of 3 months.

Within the diagnosis procedure of Ace Alzheimer Center Barcelona, all participants had a first neurological and neuropsychological visit (with the neuropsychological battery of Fundació ACE (NBACE) [24, 25] administered by one of the neuropsychologists of the team), including an accurate diagnosis to ensure that they fulfilled the BIOFACE inclusion criteria. The participants returned to the Memory Unit for an additional neurological assessment, followed by the FACEmemory visit, which was performed in another office with a psychologist who invited each patient to sit at a table with a tablet and complete the FACEmemory test with minimal supervision (only technological issues were addressed). Finally, in the same week, each participant came back for an additional neuropsychological assessment administered by a neuropsychologist (A.P.) in the same order. Thus, the neuropsychological assessment included three visits: (1) the NBACE, (2) additional neuropsychological tests to detect prodromal AD, and (3) the FACEmemory test.

Inclusion criteria were diagnosis of MCI according to the Petersen [3] criteria (including the amnestic and non-amnestic types), a Clinical Dementia Rating of 0.5, age of onset under 65, Mini-Mental State Examination (MMSE) score ≥ 24, educational level of at least elementary school, capacity to provide written informed consent, and fluent Spanish language skills. Exclusion criteria were contraindication for brain MRI, presence of an underlying medical or neurological illness that could account for cognitive impairment based on lab tests or brain imaging, major psychiatric disorder, active drug abuse, and severe auditory/visual abnormalities.

In addition to FACEmemory, the following classical memory tests were administered: the Spanish version of the Word FCSRT [11, 26], the Word List from the Wechsler Memory Scale, third edition (WMS-III) [27], and the Spanish version of the Rey-Osterrieth Complex Figure Test (ROCF) [26].

### FACEmemory and other memory test procedures

The FACEmemory test was administered via a tablet computer with voice recognition and a touchscreen, ensuring its standardized administration and immediate automatized scoring and registration of the results in an anonymous database.

As detailed elsewhere [17], the temporal sequence of FACEmemory was the following: two learning trials, a short-term memory task, and a long-term memory task that included face, name, and occupation memory recognition. The total test duration was approximately 30 min. Briefly, the first learning trial consisted of a total of 12 faces, each one associated with a name and an occupation that appeared beneath it for 8 s. The second learning trial followed the same procedure as the first one, but the 12 faces appeared in a different order. Participants were instructed to read the name and occupation appearing beneath each face aloud and to try to remember it. Then, the application asked the participants to press the red microphone button and say the name and occupation they remembered as being associated with each face. Two minutes after the second learning trial began, the application again asked the participants to say the name and occupation they remembered as being associated with each face. Finally, 20 min after the second learning trial, long-term memory assessment was initiated, which involved free recall and recognition tasks. First, the participants were instructed to recognize, from three faces, the face that had appeared in the first learning trial and to touch it. Then, the correct face appeared and the participants were asked to say the name and occupation they remembered for each face. After each answer, a screen appeared showing the correct face, and two rows below the face were three name options and three occupation options. The participants were instructed to touch the name and occupation they recognized as being associated with that face.

With regard to the Spanish version of the Word FCSRT [11], the task consists of learning 16 words that are presented on four pages. Participants were asked to read each word aloud and then say which of the words corresponded to a given semantic category. After this initial learning and encoding procedure, there were three recall trials, each preceded by a non-semantic interference task (countdown of 20 s). For each trial, participants were asked to remember as many words as possible, and then, the semantic category was provided for those items that were not recalled. The same recall procedure was repeated 20 min later.

The Word List from the WMS-III [27] was administered using a traditional procedure in which the list was presented orally in four consecutive learning trials, without using any interference list. After a 20-min delay, a free recall trial, followed by a yes/no recognition task, was administered [24].

With regard to the ROCF [26] test, a complex figure was placed in front of the participant, who was asked to copy it as accurately as possible. Then, the figure was removed from view. After a 20-min delay, the subject was asked to reproduce the figure from memory.

### Acquisition and processing of brain MRI images

Scans were acquired in a Siemens MAGNETOM VIDA 3T scanner (Erlangen, Germany) using a 32-channel head coil from Clínica Corachan (Barcelona). T1-weighted images, for the morphological and the volumetric studies, were acquired using a gradient-echo 3D MPRAGE sequence with the following parameters: TR 2200 ms, TE 2.23 ms, TI 968 ms, 1.2-mm slice thickness, FOV 270 mm, and voxel measurement 1.1 × 1.1 × 1.2 mm. To complete the acquisition, a 3D isotropic FLAIR, an axial sequence T2-weighted and an axial sequence T2*-weighted gradient recall echo were performed to detect vascular brain damage and microbleeds. All images were stored in a PACS and submitted to an automated process of deidentification.

The MRIs were processed at the neuroimaging laboratory at Ace Alzheimer Center Barcelona. After a visual inspection for artifacts, cortical and subcortical segmentation of the structural images was performed using FreeSurfer 7.2 (https://surfer.nmr.mgh.harvard.edu/). This procedure allows the segmentation of gray matter, white matter, and other substructures. A surface-based morphometry (SBM) analysis of cortical volume was performed for the total FACEmemory score variable, with age, schooling years, sex, and total intracranial volume as covariables. Surfaces were smoothed with a 10-mm FWHM kernel. Cluster-wise correction for multiple comparisons was done using a *z*-based Monte Carlo simulation with 10,000 iterations. Surface clusters were looked at with a cluster-forming threshold of *p*< 0.001 and corrected for multiple comparisons for *p*< 0.05.

### Lumbar puncture and cerebrospinal fluid collection

This protocol followed the consensus recommendations established by the Alzheimer’s Biomarkers Standardization Initiative [28]. Briefly, a lumbar puncture was performed by an experienced neurologist with the patients in a seated position and under fasting conditions. After applying local anesthesia (1% mepivacaine) subcutaneously, the neurologist obtained CSF by lumbar puncture in the intervertebral space of L3–L4. The fluid was collected passively in two 10-ml polypropylene tubes (Sarstedt Ref. 62610018). The first tube was analyzed externally for basic biochemistry (glucose, total proteins, proteinogram, and cell type and number). The second tube was centrifuged (2000×*g* 10 min at 4 °C), and the fluid was aliquoted into polypropylene tubes (Sarstedt Ref. 72694007) and stored at −80 °C until analysis. Time delay between CSF collection and storage was less than 2 h.

On the day of the analysis, the aliquots were thawed at room temperature and vortexed for 5–10 s to determine AD biomarkers in CSF. One aliquot/patient was used to determine the concentrations of Aβ1-42, Aβ1-40, t-tau, and p181-tau using chemiluminescence enzyme immunoassay (CLEIA) with the commercially available Lumipulse G™ reagents in the Lumipulse™ platform (Fujirebio, Europe) at the research laboratory of Ace Alzheimer Center Barcelona.

### The ATN groups

Using CSF and MRI biomarkers, participants were classified into three categories according to the ATN scheme [29]. Those categories were normal AD biomarkers (A-T-N-), Alzheimer’s continuum (including A+T-N-, A+T+N-, A+T+N+, and A+T-N+), and non-AD pathologic changes (-SNAP-, including A-T+N-, A-T-N+ and A-T+N+), where A refers to aggregated Aβ, T to aggregated tau, and N to MRI neurodegeneration or neuronal injury. Cut-offs from the Fundació ACE biomarker research program (FACEBREP) cohort were used to dichotomize each CSF biomarker into +/− as follows: Aβ1-42/Aβ1-40 ratio< 0.063 for A, p181-tau> 54 pg/ml for T, and presence of neurodegeneration in the MRI for N.

The N classification was obtained using a machine learning method (Naive Bayes) [30]. As a training database, the subjects’ data were selected from the ADNI database (adni.loni.usc.edu) [31] as follows. Only baseline MRI data from individuals diagnosed with dementia and a cognitively healthy status at baseline were selected, for a total of 1222 data points. The volume from the hippocampus, entorhinal cortex, middle temporal cortex, and lateral ventricles, as well as the subject’s age and estimated intracranial volume, was chosen as independent variables. Subjects diagnosed with dementia were considered to be neurodegeneration positive (N+), while cognitively healthy subjects were considered as neurodegeneration negative (N−). To test the algorithm, ADNI data was randomly spliced in two datasets with 70% of the data as the training dataset and 30% as the test dataset. The algorithm so fed was tested and showed an accuracy of 82%, sensitivity of 82%, and specificity of 80% for N classification. So, the full ADNI dataset was used to build the prior probability function. Then, the values of independent variables for the BIOFACE cohort were calculated and the N values were assessed as the dichotomization of the posterior probability function.

### Statistical analysis

Statistical analysis was performed using Statistical Package for the Social Sciences (SPSS) version 26 for Windows (version 26.0; SPSS Inc., Chicago, IL). All data were examined for normality, skew, and restriction of range.

Descriptive analyses for demographical, neuropsychological, and clinical variables were performed. Pearson’s correlation analyses were carried out between FACEmemory scores/CSF AD biomarkers and age and schooling years. Moreover, *t* test and chi-square analyses were performed to compare demographical and FACEmemory scores between participants with and without a lumbar puncture or between men and women.

Univariate and multiple linear regression analyses (the stepwise procedure) were carried out to search for traditional memory variables (delayed free recall on the Word List from the WMS-III and Word FCSRT, total free recall/learning on the Word List from the WMS-III and on the Word FCSRT, and long-term visual recall on the ROCF) correlated with the total FACEmemory score, after adjusting for age, schooling years, and sex.

In the subsample with lumbar puncture, univariate linear regression analyses with FACEmemory and each CSF AD biomarker value, adjusting for age, schooling years, and sex, were carried out. Moreover, to search for differences between ATN groups (AD, SNAP, and normal), an analysis of covariance (ANCOVA) was performed.

For all the analyses, an effect was considered significant when *p*< 0.05 and all hypotheses were tested directionally at a 95% confidence level.

## Results

### Demographic variables

The whole sample consisted of 94 participants, including 37 men and 57 women. Their mean age was 60.74 (standard deviation (SD): 3.42) years, with a mean of 12.05 (SD: 5.08) schooling years. Their mean score on the MMSE was 28 (SD: 1.50), and on the FACEmemory, their mean score was 34.15 (SD: 18.92) points.

With regard to the type of MCI, 46 were amnestic and 48 were non-amnestic. Both groups did not significantly differ in age, sex, or years of formal education. However, as expected, aMCI performed significantly worse than non-amnestic mild cognitive impairment (naMCI) on the FACEmemory total score (mean: 26.87, SD: 16.56, and mean: 41.13, SD: 18.56, respectively; *t*= −3.92, *p*< 0.001).

The subset of patients who underwent a lumbar puncture (*n*= 82) and those who did not (*n*= 12) were homogeneous on age (*t*= 0.92, *p*= 0.357), sex (*χ*^2^= 1.19, *p*= 0.276), schooling years (*t*= 0.22, *p*= 0.826), MMSE (*t*= 0.00, *p*= 1.000), and total FACEmemory score (*t*= 0.48, *p*= 0.629). With regard to the ATN groups, 24 subjects were classified as normal, 15 AD, and 43 non-AD/SNAP. Their demographic and clinical characteristics are detailed in Table 1.

**Table 1:** Demographic and clinical characteristics of the whole sample and ATN groups

	Whole sample	ATN groups		
		Normal	AD	SNAP
*N*	94	24	15	43
Sex *n* (%) men	37 (39.36)	11 (45.80)	5 (33.30)	18 (41.90)
Age in years (mean/SD)	60.74 (3.42)	59.83 (2.55)	59.83 (2.55)	60.30 (4.17)
Schooling years (mean/SD)	12.05 (5.08)	12.42 (5.49)	12.42 (5.49)	11.86 (4.39)
MMSE (mean/SD)	28.00 (1.50)	28.13 (1.57)	27.80 (1.61)	28.00 (1.50)
MCI type *n* (%) aMCI	46 (48.94)	7 (29.16)	9 (60.00)	21 (48.84)

ATN groups: (1) Normal: normal AD biomarkers (A-T-N-); (2) AD: Alzheimer’s disease continuum (including A+T-N-, A+T+N-, A+T+N+, and A+T-N+); and (3) SNAP: non-AD pathologic changes (including A-T+N-, A-T-N+, and A-T+N+), where A refers to aggregated Aβ, T to aggregated tau, and N to MRI neurodegeneration or neuronal injury

*MMSE* Mini-Mental State Examination, *SD* Standard deviation, *MCI* Mild cognitive impairment, *aMCI* Amnestic mild cognitive impairment

The total FACEmemory score was found to be weakly correlated with age (*r*= −0.22, *p*= 0.032), but not with schooling years (*r*= 0.08, *p*=0.419) or sex (*t*= 0.16, *p*= 0.872).

### FACEmemory vs traditional episodic memory tests

The performance on the memory tests administered is detailed in Table 2. Convergent validity of FACEmemory was determined based on performance on the FCSRT. Univariate linear regression analyses showed FACEmemory to be significantly associated with each individual traditional memory test (as detailed in Table 3). In a multiple linear regression analysis using classical memory tests as independent variables, adjusted by age, schooling, and sex, long-term free recall on the Word List from the WMS-III and on the FCSRT emerged as the only significant factors associated with the total FACEmemory score (*F*= 12.830, *p*< 0.001; beta= 0.42, *p*< 0.001 and beta= 0.30, *p*= 0.001, respectively).

**Table 2:** Performance on FACEmemory and the other memory tests

Test/variable	Whole sample	ATN groups			*F* (1, 79)	*P*
		Normal	AD	SNAP		
**FACEmemory**						
LN1+2	3.78 (4.17)	5.71 (4.71)*	1.80 (2.34)	3.48 (3.53)	5.381	0.006
LO1+2	11.88 (5.58)	13.67 (4.49)*	7.60 (6.74)	12.58 (5.03)*	6.803	0.002
RSN	2.62 (2.88)	4.00 (3.18)*	1.20 (1.78)	2.47 (2.55)*	5.515	0.006
RSO	7.15 (3.18)	8.42 (2.19)*	4.27 (4.04)	7.58 (2.78)*	10.173	<0.001
FR	11.91 (0.35)	12.00 (0.00)*	11.60 (0.74)	11.95 (0.21)*	7.115	0.001
RLN	2.23 (2.62)	3.29 (2.87)*	0.73 (0.96)	2.28 (2.45)*	5.252	0.007
RLO	6.50 (3.39)	8.13 (2.67)*	3.67 (3.50)	6.60 (3.27)*	9.257	<0.001
REN	8.00 (2.71)	8.88 (2.07)	7.27 (2.74)	7.74 (2.91)	2.064	0.134
REO	11.05 (1.53)	11.63 (0.87) *	10.00 (2.00)	11.23 (1.23) *	7.331	0.001
Total score	34.15 (18.92)	43.17 (16.96)*	19.27 (17.84)	35.00 (16.98)*	9.018	<0.001
**Word FCSRT**						
Total free recall	22.95 (6.47)	24.17 (5.81)*	18.07 (7.38)	23.98 (5.91)*	5.764	0.005
Total recall	38.44 (8.57)	40.50 (5.39)*	31.60 (13.29)	39.74 (7.10)*	6.586	0.002
Delayed free recall	8.05 (4.93)	8.46 (2.80)*	4.80 (4.71)	9.05 (5.78)*	4.278	0.017
Delayed total recall	12.75 (3.54)	13.92 (1.86)*	8.40 (5.38)	13.70 (2.12)*	20.788	<0.001
**ROCFT**						
Delayed recall	12.36 (5.56)	14.44 (4.58)*	8.47 (6.33)	12.67 (5.26)*	5.995	0.004
**Word list from the WMS-III**						
Delayed free recall	4.95 (2.51)	5.58 (2.19)*	3.27 (2.63)	5.48 (2.40)*	5.452	0.006
Recognition memory	20.82 (3.54)	21.46 (2.95)	19.47 (4.37)	20.95 (3.57)	1.494	0.231

*SD* standard deviation, *LN1+2* names recalled in learnings 1 and 2, *LO1+2* occupations recalled in learnings 1 and 2, *RSN* names in short recall, *RSO* occupations in short recall, *FR* face recognition, *RLN* names in long-term recall, *RLO* occupations in long-term recall, *REN* names recognized, *REO* occupations recognized, *FCSRT* Free and Cued Selective Reminding Test, *ROCFT* Rey-Osterrieth Complex Figure Test, *WMS-III* Wechsler Memory Scale-Third Edition

^*^Post hoc statistically significant differences with AD ATN group

**Table 3:** Univariate linear regression analyses with FACEmemory and each individual traditional memory test

	*β*	*F* (1, 91)	*P* value
**Word FCSRT**			
Total free recall	0.44	21.515	<0.001
Total recall	0.37	14.668	<0.001
Delayed free recall	0.48	26.940	<0.001
Delayed total recall	0.42	19.597	<0.001
**ROCFT**			
Delayed recall	0.45	23.454	<0.001
**Word list from the WMS-III**			
Delayed free recall	0.55	40.591	<0.001
Recognition memory	0.38	15.819	<0.001

### FACEmemory vs cortical volume measured by brain MRI

The SBM analysis showed the presence of four different clusters where there was a significant correlation between cortical volume and the FACEmemory total score. In the left hemisphere, there were two clusters: the first one comprised the fusiform gyrus and associative visual cortex (*p*< 0.0002), and the second one was centered in the middle temporal cortex (*p*< 0.01). The other two clusters were located in the right prefrontal cortex (*p*< 0.0008) and in the parietal angular and supramarginal gyri (*p*< 0.004).

**Fig. 1:**
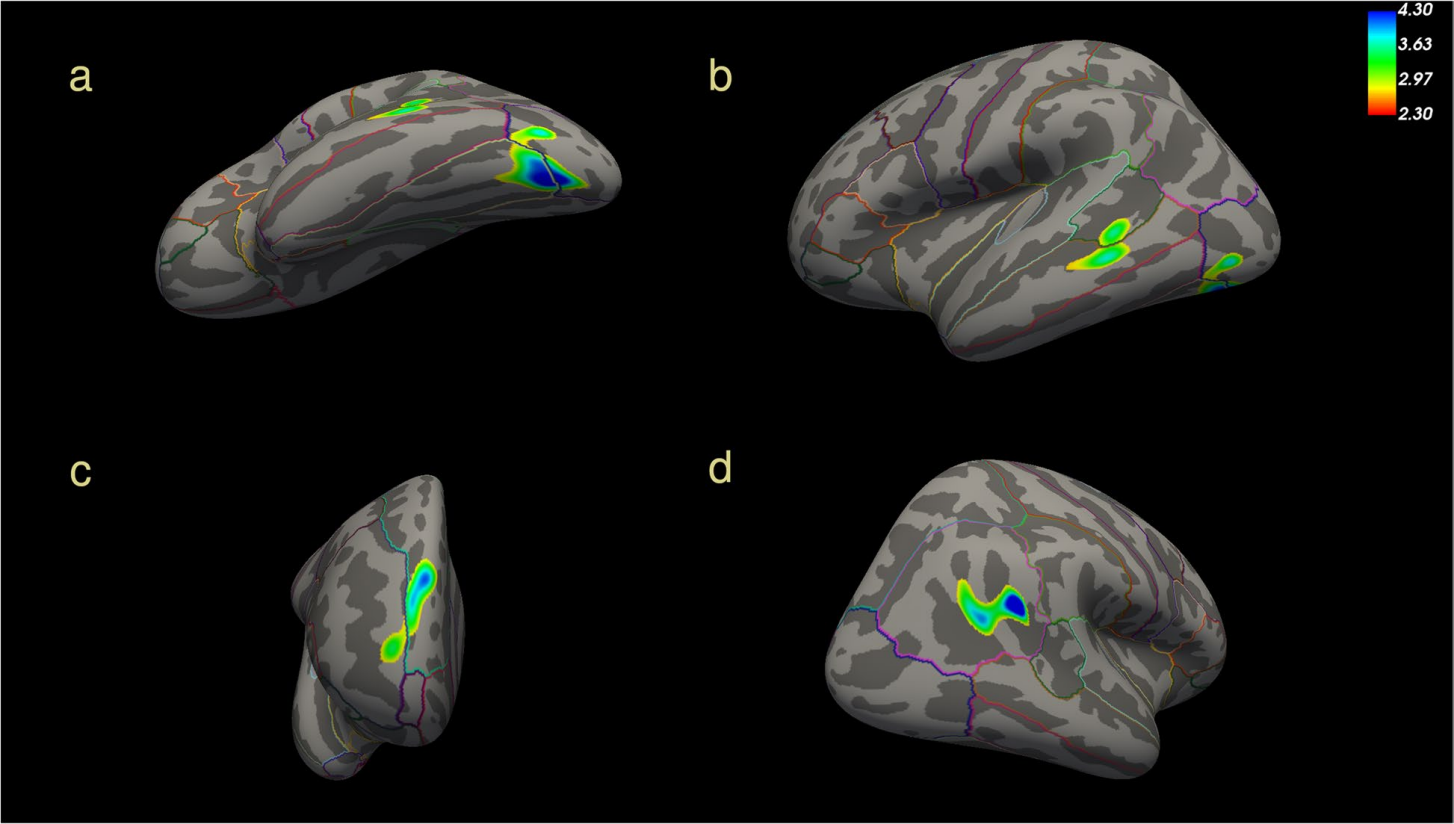
Clusters of association on left (**a**, **b**) and right (**c**, **d**) hemispheres between total FACEmemory score and cortical volume. The color map represents the value of log probabilities in the association. A worse performance on FACEmemory is associated with a lower cortical volume on each cluster

For all clusters, there was a positive correlation between memory performance and cortical volume; that is, worse performance on the FACEmemory test was associated with lower cortical volume in those areas. All the clusters are described in Table 4 and shown in Fig. 1.

**Table 4:** Cluster characteristics for SBM analysis with cortical volume and total FACEmemory score

Hemisphere	Cortical areas affected	Size (mm^2^)	Cluster wise *p* value	Brodmann areas affected	CoG Talairach coordinates (*X*, *Y*, *Z*)
Left	Fusiform, lateral occipital	745	0.0002	Associative visual cortex (19), Fusiform gyrus (37)	−31, −79, −11
Left	Middle temporal, bank of superior temporal sulcus	316	0.01	Superior temporal gyrus (22)	−62, −42, −7
Right	Superior frontal, rostral middle frontal	483	0.0008	Dorsolateral prefrontal cortex (9), anterior prefrontal cortex (10)	10, 59, 27
Right	Inferior parietal	426	0.004	Angular gyrus (39), Supramarginal gyrus (40)	46, −50, 24

### FACEmemory vs AD-related CSF biomarkers

In the subsample of 82 patients who underwent lumbar puncture, a correlation analysis among demographical variables (age, sex, and education) showed significant associations between age and the Aβ1-42/Aβ1-40 ratio (*r*= −0.35, *p*= 0.001), p181-tau (*r*= 0.32, *p*= 0.003), and the Aβ1-42/p181-tau ratio (*r*= 0.39, *p*= 0.009) levels, but not with Aβ1-42 alone (*r*= 0.04, *p*= 0.728). Sex and schooling years were not significantly associated with any CSF variable. Univariate linear regression analyses with FACEmemory and each CSF variable, covariated by age, schooling years, and sex, showed that the total FACEmemory score was moderately correlated with the CSF Aβ1-42/Aβ1-40 ratio, p181-tau, and the Aβ1-42/p181-tau ratio, but only weakly with Aβ1-42 levels (see Table 5 for details).

**Table 5:** Univariate linear regression analyses with FACEmemory and each individual CSF variable

	*β*	*F* (1, 80)	*P* value
Aβ1-42	−0.24	5.011	<0.028
p181-tau	−0.44	19.454	<0.001
Aβ1-42/Aβ1-40 ratio	0.41	16.538	<0.001
Aβ1-42/p181-tau ratio	0.37	12.473	0.001

### FACEmemory vs ATN groups

With regard to ATN analysis, when comparing the total FACEmemory score among normal, AD, and non-AD/SNAP groups, a statistically significant effect was found among groups (*F*= 9.018, *p*< 0.001). Post hoc analysis showed that the AD group differed significantly from the normal and SNAP groups (with a mean difference of 23.90 (SE: 5.64), *p*< 0.001, and 15.73 (SE: 5.14), *p*= 0.009, respectively) (for details see Table 2).

## Discussion

In a cohort of patients with EOMCI, the results of the present study show that the worse performance on the FACEmemory test was related to worse performance on traditional episodic memory tests sensitive to the AD endophenotype and was also associated with AD-related neuroimaging and CSF biomarkers. This is the first study demonstrating the convergent validity of a computerized verbal episodic memory tool with voice recognition, the FACEmemory test, and its correlation with MRI and CSF variables [29, 32, 33].

Firstly, as expected, patients with a diagnosis of aMCI performed significantly worse on the FACEmemory test than those with naMCI. With regard to the association between FACEmemory and classical memory test performances, univariate linear regression analyses showed worse performance on the computerized FACEmemory test significantly associated with visual and verbal long-term memory on classical memory tests (Word FCSRT, ROCFT, and Word List from the WMS-III). However, a multiple linear regression analysis showed that it was mainly related to worse performance on delayed free recall on the FCSRT and the Word List from the WMS-III, demonstrating that FACEmemory allows the detection of the AD endophenotype or storage impairments in episodic memory, the most important risk factor for conversion from MCI to AD dementia [21, 22, 34, 35]. The FCSRT is another memory test that enhances learning and can distinguish retrieval from storage impairment [11, 36]. Thus, our findings support that FACEmemory is sensitive to storage or hippocampal memory dysfunction [13, 37], reinforcing that it may be a suitable endophenotype of AD [5].

Cognitive approaches conceptualizing face-name associative memory have demonstrated that associating unfamiliar faces with proper names is a more complex task than other episodic memory tests because it is an arbitrary association [38]. The FACEmemory test asks the participants to learn the name and occupation of 12 unknown faces, demanding arbitrary face-name associations, which are sensitive to preclinical and prodromal AD [20, 39]. Thus, consistent with previous studies with different versions of FNAME [20, 39, 40], FACEmemory may be a suitable test to detect episodic memory impairment, which is needed for the early detection of AD [21, 22, 41].

With regard to neuroimaging MRI results, the present study found that worse execution on FACEmemory was associated with lower cortical volume in associative visual, prefrontal, and temporo-parietal areas. These brain regions are sensitive to EOAD. It is well known that medial temporal, prefrontal, parietal, and occipital cortices are significantly involved in EOAD pathology [8, 42–45], with a predominant posterior neocortical atrophy when compared to late-onset AD [8, 46]. Moreover, this finding agrees with previously published neuroimaging studies demonstrating that faces are encoded by the non-dominant (usually right) hemisphere and names and occupations are encoded by the language areas of the brain, the dominant (usually left) hemisphere [47]. To learn and recall the names and occupations of unknown faces implicates bilateral associative occipito-temporal cerebral regions with extensive connections to cortical areas, which is the specific neural function disrupted in AD [20, 47]. For this reason, FACEmemory performance requires cognitive functions highly vulnerable to AD.

Finally, worse execution on FACEmemory was found to be moderately associated with the Aβ1-42/Aβ1-40 ratio, p181-tau, and the Aβ1-42/p181-tau ratio and weakly with Aβ1-42 levels. It is well known that the Aβ1-42/Aβ1-40 ratio is more sensitive than Aβ1-42 alone to detect AD pathology, and its use is recommended when analyzing CSF AD biomarkers to improve the percentage of appropriately diagnosed MCI patients [48–50]. Consequently, the correlation found in this study with the FACEmemory test is of great value because it could reflect sensitive AD pathological changes in CSF. These results reinforce studies demonstrating an association between amyloid burden, either with PET imaging or CSF, and performances on traditional (the original FNAME [20], the Spanish version of FNAME [39]) and computerized (PAL CANTAB [5], FACEmemory [17]) episodic memory tests. FACEmemory scoring was also found to be related to p181-tau and the Aβ1-42/p181-tau ratio, supporting the idea that biomarkers of neurodegeneration are closely related to clinical symptoms [29], taking into account that the Aβ1-42/p181-tau ratio includes both AD biomarkers.

With regard to ATN classification [29], when performance on FACEmemory® was compared between 24 EOMCI subjects with normal AD biomarkers (A-T-N-), 43 SNAP (including A-T+N-, A-T-N+, and A-T+N+), and 15 AD continuum (A+T-N-, A+T+N-, A+T+N+, and A+T-N+), a significant effect was found among groups. A mean difference of 23.9 points in the FACEmemory score was detected between normal and AD ATN groups. These findings support that this test seems to be a diagnostic tool that reflects AD pathological changes [29, 51]. Moreover, the AD ATN group had the highest percentage of patients with aMCI and performed considerably lower on most of the memory tests compared to the other ATN groups. However, the normal and SNAP ATN groups also included memory-impaired cases (29.16% and 48.74%, respectively). Although the original FNAME was created to detect preclinical AD [20], the results of the present study suggest that FACEmemory will also allow the detection of memory-impaired cases with other etiologies. In fact, EOMCI is not always related to prodromal AD, as many other underlying conditions (i.e., neurodegenerative, psychiatric) can be associated with this MCI category [4].

In our opinion, the findings of the present study are important because they pave the way for a novel automatized method to detect AD and non-AD MCI in younger people. Consequently, FACEmemory may be a suitable complex episodic memory test to be used in remote settings, such as in the context of clinical trials, in a standardized and easy way. Moreover, the complexity of FACEmemory makes it a potential complement to classical memory testing in clinical practice of patients with very early cognitive impairment.

Further longitudinal studies will be needed to determine whether lower baseline scores on FACEmemory are related to an increased risk of developing AD or another type of dementia.

### Limitations

We acknowledge that our study has several limitations. First, the study design is cross-sectional. However, this is the first step of the BIOFACE project, in which all participants are being followed up annually with clinical and biomarker assessments. Secondly, not all recruited participants gave their consent for a CSF sample, but we recognize that some of them were reluctant to undergo a lumbar puncture due to it being an invasive, not completely innocuous process that can cause pain or discomfort to the patient. Thirdly, ADNI data was used as a training dataset for the neurodegeneration classification algorithm. ADNI data contains subjects with a wider age range which are older, on average, than the current dataset. Although the machine learning algorithm chose the proper prior distribution for any involved variable, it automatically discarded a considerable number of subjects from the training database. The use of a fully representative dataset for the current sample could be worthy of study. Finally, although all participants have been accurately characterized with an exhaustive cognitive neuropsychological assessment and multimodal AD biomarkers, the small sample size may prevent us from making robust conclusions. However, it should be noted that this is a single-center study and patients with an early-onset cognitive impairment are less frequently derived to a memory clinic than those with late-onset cognitive impairment.

## Conclusion

The results of this study confirm that FACEmemory is a promising tool for detecting memory deficits related to underlying AD, but also for detecting memory-impaired cases with other etiologies. Our findings suggest that FACEmemory scoring can detect the endophenotype of AD, which is a hippocampal memory impairment. It is also associated with AD-related changes in MRI and CSF in patients with EOMCI, an MCI subtype in which a diagnosis of prodromal AD usually takes longer, probably due to a lower suspicion of a neurodegenerative condition in younger people. However, FACEmemory can also detect memory-impaired cases with a non-AD etiology. The computerized FACEmemory tool might be an opportunity for facilitating early detection of *memory impairment* in younger people than 65, who have a growing interest in new technologies.

## Data Availability

Data used can be requested through the corresponding author.

## Abbreviations

AD: Alzheimer’s disease
aMCI: Amnestic mild cognitive impairment
ANCOVA: Analysis of covariance
CSF: Cerebrospinal fluid
EOAD: Early-onset Alzheimer’s disease
EOMCI: Early-onset mild cognitive impairment
FACEBREP: Fundació ACE biomarker research program
FCSRT: Free and Cued Selective Reminding test
FNAME: Face-Name Associative Memory Exam
FR: Face recognition
LN1+2: Names recalled in learning 1 + 2
LO1+2: Occupations recalled in learning 1 + 2
MCI: Mild cognitive impairment
MRI: Magnetic resonance imaging.
MMSE: Mini-Mental State Examination
N+: Neurodegeneration positive
N−: Neurodegeneration negative
naMCI: Non-amnestic mild cognitive impairment
NBACE: Neuropsychological battery of Fundació ACE
REN: Names recognized
REO: Occupations recognized
RLN: Names in long-term recall
RLO: Occupations in long-term recall
ROCFT: Rey-Osterrieth Complex Figure Test
RSN: Names in short recall
RSO: Occupations in short recall
SBM: Surface-based morphometry
SD: Standard deviation
SNAP: Non-AD pathologic changes
SPSS: Statistical Package for the Social Sciences
WMS-III: Wechsler Memory Scale, third edition
